# CRISPR-based strategies for sample-to-answer monkeypox detection: current status and emerging opportunities

**DOI:** 10.1088/1361-6528/ad892b

**Published:** 2024-11-04

**Authors:** Md Ahasan Ahamed, Anthony J Politza, Tianyi Liu, Muhammad Asad Ullah Khalid, Huanshu Zhang, Weihua Guan

**Affiliations:** 1 Department of Electrical Engineering, Pennsylvania State University, University Park, PA 16802, United States of America; 2 Department of Biomedical Engineering, Pennsylvania State University, University Park, PA 16802, United States of America

**Keywords:** monkeypox, nucleic acid, CRISPR, Cas12, RPA, point-of-care

## Abstract

The global health threat posed by the Monkeypox virus (Mpox) requires swift, simple, and accurate detection methods for effective management, emphasizing the growing necessity for decentralized point-of-care (POC) diagnostic solutions. The clustered regularly interspaced short palindromic repeats (CRISPR), initially known for its effective nucleic acid detection abilities, presents itself as an attractive diagnostic strategy. CRISPR offers exceptional sensitivity, single-base specificity, and programmability. Here, we reviewed the latest developments in CRISPR-based POC devices and testing strategies for Mpox detection. We explored the crucial role of genetic sequencing in designing crRNA for CRISPR reaction and understanding Mpox transmission and mutations. Additionally, we showed the integration of CRISPR-Cas12 strategy with pre-amplification and amplification-free methods. Our study also focused on the significant role of Cas12 proteins and the effectiveness of Cas12 coupled with recombinase polymerase amplification (RPA) for Mpox detection. We envision the future prospects and challenges, positioning CRISPR-Cas12-based POC devices as a frontrunner in the next generation of molecular biosensing technologies.

## 1. Introduction

The Monkeypox Virus (Mpox) is an emerging global health concern due to its ability to spread rapidly and cause outbreaks [1, 2]. The genetic complexity of Mpox, characterized by its intricate genome with inverted tandem repeats, open reading frames, and hairpin loops, along with the presence of multiple virulent variants, presents challenges for timely and accurate detection [3]. Since its discovery in 1958, Mpox outbreaks have primarily occurred in West Africa and the Congo basin. African rodents or small mammals like those in the Funisciurus and Heliosciurus genera are suspected to be natural reservoirs [4]. However, the mortality rate of Mpox varies depending on the clade. In Africa, case fatality rates range from 1% to 10%, which is higher among children [5]. The virus has two main clades: Clade I (Congo Basin), with over 10% fatality, and Clade II (West African), including subclades IIa and IIb, with less than 1% fatality [6]. The 2022 outbreak involved the more lethal Clade IIb strain, first identified in Massachusetts, USA, with a concerning fatality rate of around 10% [7, 8]. As of 20 November 2023, the World Health Organization reported 91 788 laboratory-confirmed cases, 660 probable cases, and 167 deaths across 116 countries [9, 10]. The Centers for Disease Control and Prevention (CDC) closely monitors worldwide outbreaks, reporting 92 048 confirmed cases across 117 countries, primarily associated with the strain initially detected in the USA during the 2022 Mpox outbreak [11]. This situation underscores the urgency of effective disease control, where point-of-care (POC) devices play a crucial role. Their quick detection capability is vital for timely treatment, containment, and implementation of isolation measures, particularly in regions with limited healthcare resources. Additionally, these devices streamline identifying individuals who most need vaccinations, ensuring efficient and targeted distribution of vaccines [12]. Therefore, it is crucial to develop reliable, rapid, and readily accessible POC testing devices to manage and control Mpox disease effectively.

The drive for better POC methods has accelerated the development of sophisticated nucleic acid testing (NAT) platforms. The quantitative polymerase chain reaction (PCR), the gold standard in the field of POC, is highly regarded for its straightforward application, reliability, effective performance, user-friendliness, and widespread availability [13]. However, PCR requires thermocycling; PCR has 2 main steps for thermal operation: initial denature (95 °C, 20 sec) and combined annealing/extension (60 °C, 30 sec) [14]. Thermocycling in PCR requires precise temperature control and cycling through different temperatures to achieve denaturation, annealing, and extension of DNA, which can be bulky, expensive, and dependent on a stable power supply. These requirements limit the portability and simplicity of PCR, making it less suitable for POC diagnostics. To solve this problem, the development of isothermal amplification technology such as loop-mediated isothermal amplification (LAMP) [14], recombinase polymerase amplification (RPA) [9], and nucleic acid sequence-based amplification (NASBA) [15] offers practical solutions for the quick and effective detection of NAT in POC. However, these methods often have drawbacks, such as unspecific amplification and a tendency for false positives [16]. Recent innovations in clustered regularly interspaced short palindromic repeats (CRISPR) based NAT detection, like SHERLOCK, HOLMES, DETECTOR, and Cas12aVDet techniques, have significantly improved their specificity, eliminated the issue of false positive and established itself as a core technology for next-generation nucleic acid detection [17–20]. CRISPR-based methods predominantly utilize optical detection, employing reporters with fluorescent, bioluminescent, or colorimetric signals, often incorporating Förster resonance energy transfer (FRET) [21–23]. However, recent advancements in integrating CRISPR with electrochemical [24] and nanopore sensors [9, 25] have opened new avenues for POC applications. Therefore, there is an unmet challenge to developing a fast, rapid turnaround, and field-deployable POC device to detect pathogens such as Mpox using CRISPR-Cas12 assay to enhance sensitivity and specificity.

In this work, we present the current landscape of CRISPR to detect Mpox using POC systems. Initially, we delve into the origin, spread, genetic makeup, symptoms, treatments, and emerging diagnostic methods of the Mpox virus. Subsequently, we explore how CRISPR technology has transitioned from a gene-editing tool to a diagnostic resource, focusing on the role of Cas12 and crRNA in specifically identifying Mpox and other viruses and showing both amplification-free and pre-amplification strategies in Mpox detection. Further, we draw comparisons between CRISPR-based POC diagnostics and traditional methods for Mpox detection. Finally, our review shows the insights, providing a perspective on the future direction and potential advancements in CRISPR-based POC diagnosis.

## 2. Mpox virus

### 2.1. Source, Propagation chain, Signs and Symptoms of Mpox virus

**Figure 1.**
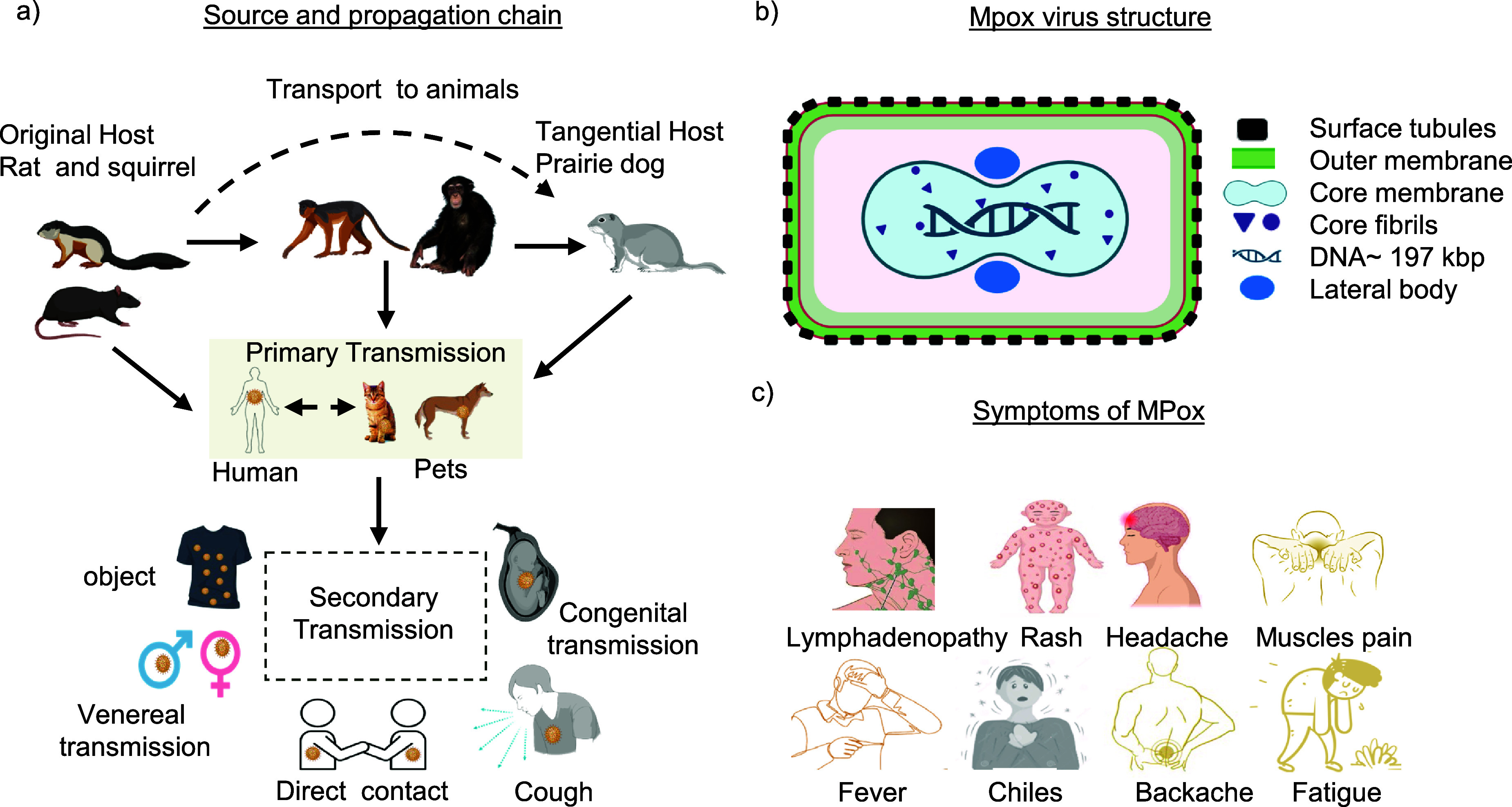
Source and transmission Cycle of Mpox virus. (a) Source and transmission cycle from animal to human and human to human. The upper section shows African rodents and squirrels transmitting the virus to humans and nonhuman primates via contact with lesions. Solid arrows indicate confirmed routes; dashed arrows suggest possible pet transmission. The dashed box highlights human-to-human spread, including respiratory droplets, contaminated items, skin contact, sexual, and possible congenital transmission. Reproduced from [29]. CC BY 4.0. (b) The Mpox virus structure, showcasing its core, membrane, lateral body, surface tubules, and nucleocapsid, with a double-concave dumbbell shape and outer lipoprotein layers. Reproduced from [30], with permission from Springer Nature. (c) Highlights the onset of Mpox with systemic symptoms like chills, muscle pain, and lymphadenopathy, accompanied by close-up views of the early skin lesions at the inoculation site. Reproduced from [31]. CC BY 4.0. Figures are drawn using Biorender.

Mpox, a zoonotic disease, is transmitted from animals to humans, with potential animal reservoirs including various mammals native to Africa, and the expansive 2022 outbreak affecting the man-sex-with-man community primarily [4, 26]. The epidemic predominantly spreads through human-to-human contact via respiratory droplets, contaminated objects, and contact with infected lesions [27]. High viral loads in bodily fluids [28] and evidence from clinical swabs suggest that sexual transmission significantly propagates the disease; details are shown in figure 1(a) [29]. The upper section of the figure delineates traditional zoonotic transmission pathways, with African rodents and squirrels serving as reservoir hosts. These species can transmit the virus to incidental hosts, including humans and nonhuman primates such as monkeys and apes, via direct contact with infectious lesions. Solid arrows represent confirmed transmission routes, while dashed arrows suggest potential, yet unconfirmed, pathways to household pets. The human-to-human secondary transmission, highlighted in the dashed box at the bottom part of the figure, includes established pathways through respiratory droplets, contact with contaminated items, direct skin contact, sexual transmission, and possible congenital transmission from pregnant individuals to their unborn children. The middle shaded part underscores the primary zoonotic transmission, while the bottom part of figure 1(a) indicates the secondary transmission among humans, capturing the multiplicity and complexity of MPXV spread.

Mpox is an Orthopoxvirus characterized by a brick-shaped structure and a large linear double-stranded DNA genome [32–34]. Observed through electron microscopy, it has a distinct morphology, typically 200–250 nm in length and a width of 140–260 nm [30], with a lipoprotein envelope and surface tubules [34, 40], which is intricately depicted in figure 1(b). The virus genome encodes all proteins necessary for replication and mRNA translation within the host cell cytoplasm [35]. Though poxviruses generally exhibit a bulky structure due to their protein coating, the specific receptor facilitating the entry of Mpox into host cells remains unidentified [34].

Mpox, entering through oropharyngeal or dermal routes, leads to systemic symptoms and a multi-stage red rash over 2–4 weeks, resolving in scarring about 3–4 weeks post-onset [36, 37]; the disease progression includes fever 101 °F to 105 °F, lymphadenopathy, and evolution of skin lesions from macules to pustules [41]. Other possible symptoms can include headaches, muscle pains, nausea, vomiting, profound tiredness, and a general sense of exhaustion, which typically emerge within one to three weeks [31, 38, 42], as shown in figure 1(c). These symptoms are particularly severe for unvaccinated, pregnant, and pediatric patients with weaker immune defenses [31]. Ongoing research is crucial for vaccine development and disease management to navigate these immune pathways and curb Mpox transmission [38, 39].

### 2.2. Genomic sequence and sample type of Mpox virus

**Figure 2.**
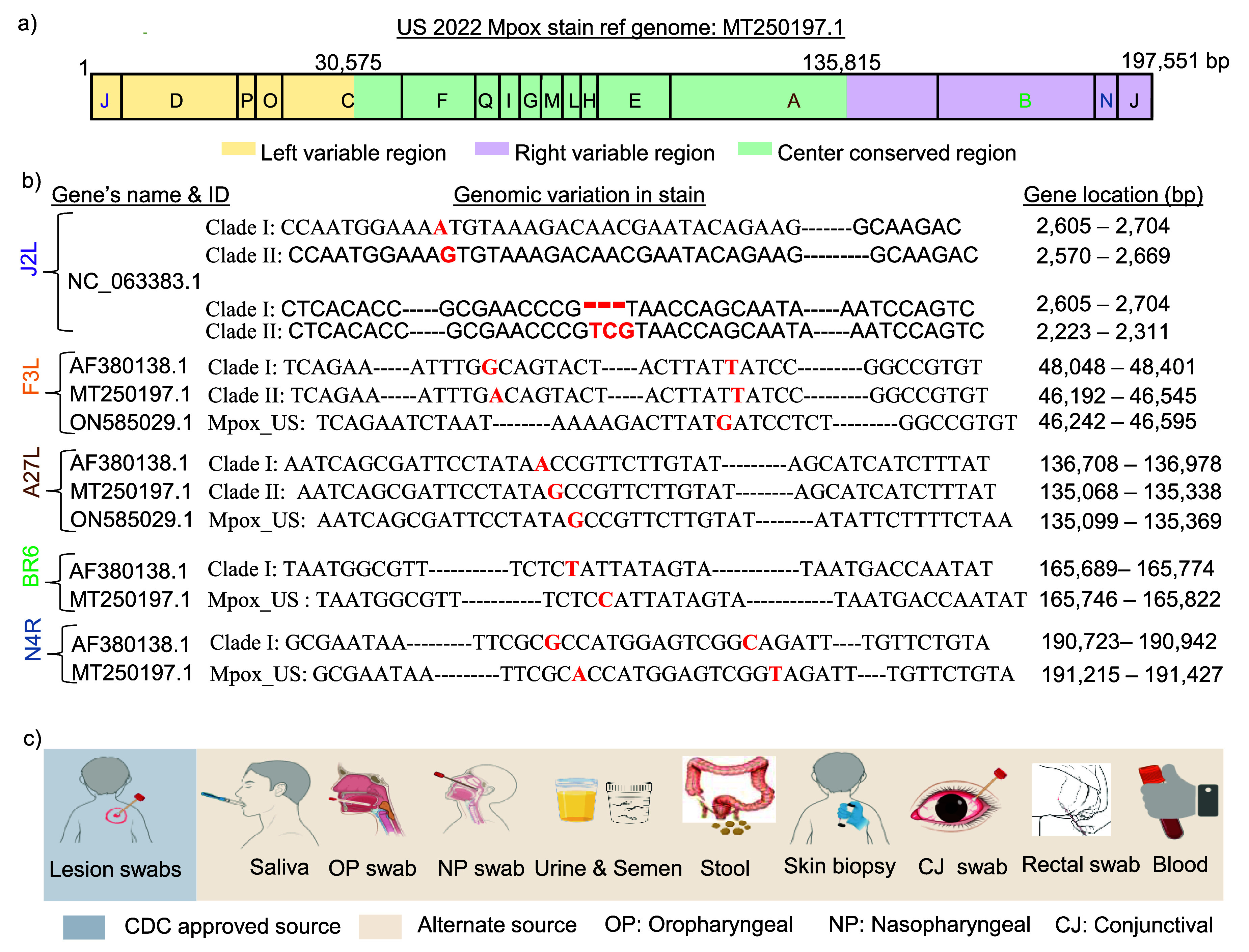
Genomic sequence and sample collection of Mpox virus. (a) Displays the 197 kb genome of Mpox, a linear DNA sequence with essential structural and enzymatic elements and variable terminal sequences for pathogenicity and host interaction capabilities. [39] John Wiley & Sons. © 2024 Araf *et al*. (b) Highlighting specific genes and mutations identified across different strains, which might be color-coded to show clade variations. (c) CDC Approved *Source*: Shows swab collection from skin lesions and saliva, preferred for Mpox diagnosis. Alternative *Sources*: Depicts collection from various sites, including nasopharyngeal, oropharyngeal, and more, as secondary diagnostic options. Figures are drawn using Biorender.

The Mpox genome, which is linear and double-stranded, spans approximately 197 kbp and codes for an estimated 200 proteins [39]. As outlined in figure 2(a), this genome is restricted to a central conserved region. Variable terminals are responsible for mutation. Surface proteins like A27L (ATI), B6R (EEV), F3L, J2L (TNFR), and N4R govern the virulence of Mpox, replication capability, and assembly [43]. Mpox, enabling it to mutate and potentially adapt to human hosts, a process underscored by the activity of the APOBEC3 enzyme [34]. This variability has been linked to disease transmission and severity variations. Figure 2(b) shows detailed genomic variations within the Mpox virus across different clades and US 2022 strains, showcasing specific gene mutations and their locations in base pairs (bp). This sort of genomic mapping is crucial for identifying strain-specific characteristics and designing the assay. It is best to use conserved regions for testing specificity, while variable regions are more suitable for testing mutated clades.

Mpox diagnostic samples, as depicted in figure 2(c), are most effectively obtained from lesion swabs (CDC-approved) due to the high viral load. Saliva is also a viable sample, offering safer and more comfortable collection options [44–46]. Oropharyngeal, nasopharyngeal swabs, skin biopsy, and blood or serum are typically used as alternate sources for virus identification because they usually contain less DNA. The diagnostic potential of bodily fluids like urine, semen, and rectal or vaginal secretions is under investigation, with proper refrigeration or freezing of samples critical for maintaining their integrity during transport to laboratories for analysis [28]. Table 1 summarizes all types of samples used in virus detection, their sensitivity, viral load interquartile range (IQR), collection difficulty level: Easy (E), Medium (M) and Diffucult (D) and minimum (Min) volume required to start the test.

**Table 1.** Summary of samples type used for Mpox detection. Reprinted from [28], Copyright (2022), with permission from Elsevier.

Sample type	Min Vol (mL)	Viral load IQR (onset)	Sensitivity/Positivity (%)	Collection difficulty	Storage lifetime days	References
Saliva	1	7–11.3	88–100	E	Refrigerate samples at 2 °C–8 °C or freeze them at −20 °C within an hour; store them long-term at −70 °C [28].	[47]
Blood	9	4.0	24–43	M		[45, 48, 49]
Serum	⩾0.5		52	D		[28]
Skin biopsy	0.5	4–10		D		[50]
Lesion swabs	1	6.5–8.2	92–100	E		[28, 48, 49, 51]
Oropharyngeal swab	3	2.9–5.8	64–76	M		[28, 45, 48, 49, 52]
Nasopharyngeal swab	3	6.0–12.0	43	M		[48, 50]
Rectal swab	3	2.9–7.5	77	M		[49, 52]
Semen	1	2.9–4.7	67–85	M		[49]
Urine	50	4.0–10.0	28	E		[48, 50, 53]

^a^E- Easy, M- Moderate, D- Difficult, IQR- Interquartile range.

### 2.3. CDC/Food and Drug Administration (FDA)-approved methods, protocols, treatment, and medicine

**Figure 3.**
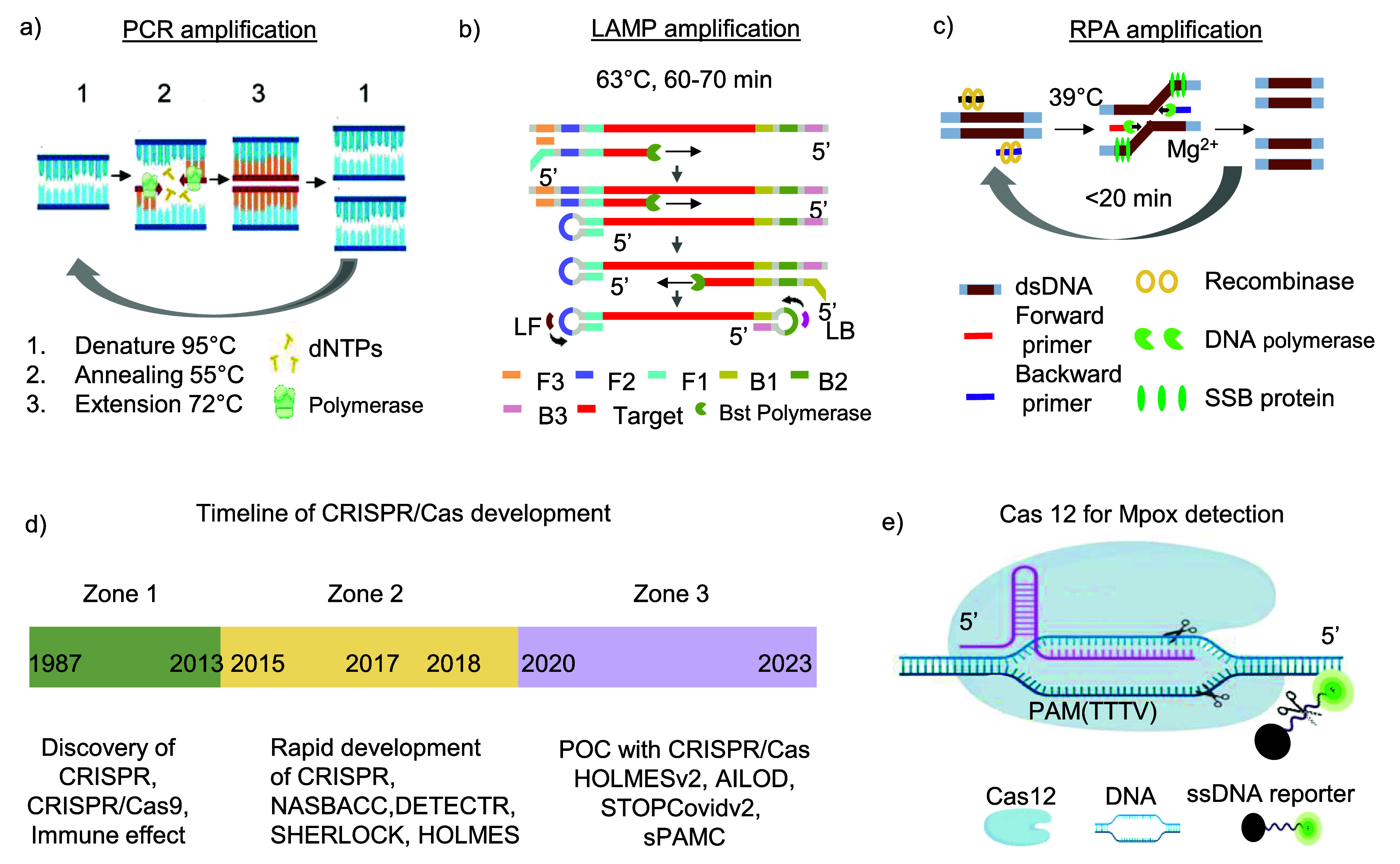
Laboratory diagnosis method of Mpox detection. (a) PCR is depicted with its core components, including DNA primers, dNTPs, polymerase, and a thermal cycler executing the denaturation, annealing, and extension cycles. (b) LAMP is shown, highlighting its mechanism with loop structures and amplification steps. Reproduced from [57]. CC BY 4.0. (c) A schematic of an RPA process showing primers and proteins. Reprinted from [9], Copyright (2024), with permission from Elsevier. (d) The timeline traces the significant developments in CRISPR/Cas technology from its origins in 1987 to its future projections in 2023. It highlights the evolution from basic gene-editing mechanisms to sophisticated *in vivo* applications and POC diagnostics. Reproduced from [62]. CC BY 4.0. (e) The essential components and mechanisms of the Cas12 system are characterized by its DNA targeting capability, using a crRNA and relying on a PAM sequence (TTTV) for recognition before cleaving the ssDNA. Notable is the collateral cleavage of a non-targeted ssDNA molecule, depicted with a green fluorophore, which is integral to its diagnostic application. Figures are drawn using Biorender. Reprinted from [63], Copyright (2023), with permission from Elsevier.

The CDC supports a US FDA-approved PCR test for detecting Mpox virus [54]. This PCR protocol is detailed in CDC guidelines [55], with figure 3(a) illustrating the PCR reaction mechanism for detecting Mpox involves cyclic temperature changes: denaturation (heating DNA to separate strands), annealing (cooling for primer attachment), and extension (raising temperature for new DNA synthesis), thereby exponentially amplifying the target DNA [47, 56, 57]. This process, noted for its high accuracy and sensitivity, uses fluorescence-based methods or gel electrophoresis for DNA quantification and analysis [58]. For testing, the CDC recommends using dry swabs or swabs in viral transport media from lesions, avoiding media for bacterial preservation [59]. Testing is advised for individuals with rashes resembling Mpox or those potentially exposed to the virus [60]. The CDC also provides specific sample storage, transport, and handling guidelines to maintain sample integrity and meet testing eligibility [61].

Regarding treatment, although no specific antiviral medication is officially approved for Mpox, drugs initially developed for smallpox have been employed in treating severe cases of Mpox. Treatment is essential for those at high risk, such as individuals with weakened immune systems, pregnant individuals, or young children. Vaccines like JYNNEOS and ACAM2000, offering cross-protection against Mpox, are recommended for high-risk groups. Other medications like Tecovirimat (TPOXX), Brincidofovir (Tembexa), and Vaccinia Immune Globulin Intravenous may be considered for treating Mpox [64, 94].

### 2.4. Emerging diagnostic methods for Mpox

PCR is a reliable, high-accuracy, and high-sensitivity method for DNA amplification and is considered the ‘gold standard’ for detecting Mpox [57, 65]. PCR with digital droplet microfluidics offers an efficient method for Mpox detection, ideal for use in POC devices [66]; refer to table 2. Given the need for precise thermal cycling in PCR, LAMP and RPA have been considered emerging diagnostic methods over the last two decades, offering alternative approaches in POC settings.

**Table 2.** Summary of the reported diagnosis methods of Mpox.

Diagnostic methods	Target gene/protein and length (bp)	Genebank source	Readout	Lod	Total time (min)	Dynamic range log(fold)	References
PCR	E9L	L22579.1	Fluorescence	12.5 cp *μ*l^−1^	43.3	5	[67]
PCR	B6R	L22579.1	Fluorescence	10 cp *μ*l^−1^	30.5	5	[67]
PCR	J1L, 125	ON631963.1	Fluorescence	100 cp *μ*l^−1^	21.6	0.9	[47]
PCR	F3L, 79	AF380138	Fluorescence	20 cp *μ*l^−1^	66.25	6	[68]
PCR	C3L		Fluorescence	40.4 cp *μ*l^−1^	24.75	8	[69]
PCR	G2R		Fluorescence	3.5 cp *μ*l^−1^	24.75	8	[69]
PCR	B7R		Fluorescence	50 cp *μ*l^−1^	60		[70]
PCR	F3L, 107	AF380138	Fluorescence	50–250 cp *μ*l^−1^	17.75	6	[65]
	N3R, 139						
PCR	Mpox-UK_P2	MT903344.1	Fluorescence	119 cp *μ*l^−1^	32.5	8	[71]
LAMP	N4R; 499	ON602722.2	Fluorescence	2 cp *μ*l^−1^	60	5	[72]
LAMP	A27L; 500	AF380138	Fluorescence	20 cp *μ*l^−1^	55	6	[73]
LAMP	D14 L, ATI	AB371721.1	Turbidity	100 cp *μ*l^−1^	>60	4	[74]
LAMP	A4L, N1R	NC_063383.1	Fluorescence	12.5 cp *μ*l^−1^	<60		[75]
LAMP	ATI	MT903346.1	Lateral flow biosensor	5 cp *μ*l^−1^	<57	5	[76]
LAMP	D14L, ATI	KP84947.1, MT903346.1	Lateral flow biosensor	5 cp *μ*l^−1^	55	5	[77]
LAMP	ATI	OP150923.1	Fluorescence	25 cp *μ*l^−1^	50	3	[78]
RPA	G2R, 300	DQ011153	Fluorescence (S- 95%, Sp-100%)	16 cp *μ*l^−1^	10	4	[79]
RPA	G2R		Lateral flow biosensor	1 cp *μ*l^−1^	20–30	5	[80]
CRISPR/Cas12a	F8L; 3020	AF380138.1	Fluorescence biosensor	4.7 cp *μ*l^−1^	90	10	[81]
RPA-CRISPR/Cas12a	N3R N4R		Fluorescence	10^8^ cp *μ*l^−1^	30		[66]
RPA-CRISPR/Cas12a	E9L; 412		Fluorescence	1 cp *μ*l^−1^	30		[82]
RPA-CRISPR/Cas12a	ATI; 392	DQ011156.1	Fluorescence	5–10 cp *μ*l^−1^	60	2	[83]
	D14L; 651	KJ642613.1					
RPA-CRISPR/Cas12a	B7R; 465	NC_063383.1	Fluorescence	13.5 cp *μ*l^−1^	35	6	[84]
LAMP -CRISPR/Cas12b	D14L; 221	KP849471.1	Nano-particle	10 cp *μ*l^−1^	60	4	[85]
	ATI; 202	MT903346.1					
RPA-CRISPR/Cas13a	F3L	NC_003310.1	Fluorescence	1 cp *μ*l^−1^	45	6	[86]
	B6R						
RPA-CRISPR/Cas12a	G2R		Fluorescence	1 cp *μ*l^−1^	30	6	[80]
RPA-CRISPR/Cas12a	F3L; 186	ON568298 928 902	Fluorescence (S- 95.8%, Sp-100%)	5 cp *μ*l^−1^	35	5	[87]
	B6R; 204						
RPA-CRISPR/Cas12a	F3L		Fluorescence	2 cp *μ*l^−1^	55	3	[88]
RPA-CRISPR/Cas12a	N3R; 530	ON563414.3	Fluorescence	1–10 cp *μ*l^−1^	30		[66]
	N4R; 1313						
RPA-CRISPR/Cas12a	D14L; 650	KP849471.1	Fluorescence	10 cp *μ*l^−1^	55	4	[88]
	ATI; 391	MT903346.1					
RPA-CRISPR/Cas12a	F3L; 117	ON563414.3	Electronic	16 cp *μ*l^−1^	55	5	[9]
RPA-CRISPR/Cas13a	F3L; 117	OP890390.1	Fluorescence	2.5 cp *μ*l^−1^	15	3	[89]
CRISPR/Cas12b—gFET	F3L	ON563414.3	g-FET	1.66 cp *μ*l^−1^	20	7	[90]
	B6R						
Electrochemical (LSG-AuNS)	A29L Protein		EIS	7.8 × 10^−3^ PFUml^−1^	15	5	[91]
Optoelectronic (MOS_2_@Au-AuICA)	A29L Protien		SERS	0.002 ng ml^−1^	20	1.69	[92]
Optoelectronics (MOS_2_@QDsICA)	A29L Protien		SERS	0.0024 ng ml^−1^	15	4.3	[93]

S- sensitivity, Sp- specificity, gFET- graphene field-effect transistor, Plaque-forming units per milliliter (PFUml^−1^), SERS- surface-enhanced Raman scattering, EIS- Electrochemical impedance spectroscopy.

LAMP is a rapid DNA isothermal amplification method (60–70 min) for Mpox detection. LAMP operates at a constant temperature between 61 °C to 65 °C and requires 6 primers set. In figure 3(b), the mechanism of LAMP is discussed with 6 primer sets [95]. Further sensitivity and specificity were improved by OSD reporters, STEM primers, and Swarm primers [96, 97]. However, LAMP needs many primers and is not useable for short-size targets. RPA is another enzyme-based amplification technique that detects Mpox quickly. It uses three enzymes: single-stranded binding protein, polymerase, and two primers like PCR [9]. The mechanism of RPA is discussed in figure 3(c). Isothermal amplification has the problems of non-specific amplification, false positives, cross-dimerization, and non-linearity of the assay [98]. The readout of LAMP and RPA is similar to PCR [99]. However, RPA and LAMP eliminate the need for thermal cycling [9]. The cost and availability of RPA kits and reagents can be a barrier, particularly in low-income regions where Mpox spreads quickly [100]. In table 2, we summarize all diagnosis methods related to Mpox detection.

Apart from the amplification assays, serological tests are used to detect Mpox, such as enzyme-linked immunosorbent assay (ELISA), plaque reduction neutralization testing, lateral flow assays (LFA), hemagglutination inhibition, immunofluorescence assay (IFA), complement fixation, and electrochemical or optical biosensors [99, 101]. Dubois *et al* investigated how unconjugated peptide combinations enhance the detection capability of their ELISA assay for later-stage Mpox infection [102]. However, Cross-reactivity among orthopoxviruses and less sensitivity significantly hamper the accuracy of serological diagnostic methods for mpox [99]. Nanopore-based next-generation sequencing, mainly using the MinION device, is a method for efficiently sequencing Mpox genomes from clinical samples [103]. Wastewater-based epidemiology is a novel technique that involves analyzing raw wastewater for biomarkers and pollutants, providing crucial data on community exposure to various environmental hazards [104]. A rapid and sensitive Mpox-detecting electrochemical sensor on paper was developed using laser-scribed graphene and gold nanospheres, capable of detecting the A29L glycoprotein with a LOD of 3.0 × 10^−16^ g ml^−1^ [91]. Another study reported a 3D multilayered molybdenum disulfide (MoS_2_) and gold nanoparticles (AuNPs) based surface-enhanced Raman scattering (SERS) dual-signal immunochromatographic assay (ICA) assay for ultrasensitive Mpox antigen detection [92]. This method showed superior performance over traditional AuNP-based colorimetric ICA assays and ELISA regarding sensitivity and testing time. Additionally, a 3D nanostructure of MoS_2_ with quantum dot shells was utilized for POC Mpox antigen detection [93]. The gold nanoparticle-based lateral flow biosensor (AuNP-LFB), a paper-based diagnostic platform, is highly regarded as an ideal tool for POC testing due to its user-friendly operation [105, 106]. These developments are compared in table 2. Despite the benefits of nanomaterial and nanostructure-based serological and POC biosensor devices, they do not match the specificity of NAT-based methods, which excel in Mpox detection when integrated with CRISPR [92, 93].

## 3. CRISPR technology and its use as a diagnostic tool

The CRISPR-Cas system, adapted from a bacterial defense mechanism, is now a vital tool in virus detection, including Mpox. It uses engineered crRNA sequences with enzymes like Cas12 and Cas13 to target and cleave specific DNA or RNA sequences, offering high specificity and sensitivity [107]. Despite its lower direct detection sensitivity, CRISPR’s effectiveness increases with amplification techniques like PCR, RPA, and LAMP. Methods such as SHERLOCK [18], HOLMES [108], and DETECTR [17, 109] exemplify its diagnostic applications. Additionally, integrating CRISPR with technologies like Field Effect Transistors (FET) and nanopore sensors enhances its utility in POC diagnostics. The CRISPR-based diverse applications extend beyond diagnostics to gene editing and regulation, making it a versatile tool in molecular biology and genetics, particularly useful for identifying pathogens and personalized medicine.

### 3.1. Fundamentals of CRISPR technology and its components

In the past decade, CRISPR systems (molecular scissors), particularly Cas9, Cas12, Cas13, and Cas14 enzymes, have dramatically transformed into molecular diagnostics from gene editing [62, 63]. Figure 3(d) shows the evolution of CRISPR [110]. Initially part of a bacterial defense mechanism, these systems have been repurposed to precisely target and cleave specific nucleic acid sequences. Cas9, known for its ability to cut double-stranded DNA accurately, has become a fundamental tool in gene editing due to its efficiency and specificity [111]. It operates by being guided to specific DNA sites, requiring an ‘NGG’ protospacer adjacent motif (PAM) for accurate targeting [63].

Similarly, Cas12 has gained prominence for its ability to target both ss/dsDNA without tracrRNA, making it highly versatile and helpful in various DNA amplification methods for diagnostics. This versatility is enhanced by its catalytic efficiency, ranging from 0.07 to 17 s^−1^, and its preference for a ‘TTTV’ PAM sequence. Additionally, Cas12 is recognized for its significant protein size range of 9–15 k amino acids (aa), making it particularly useful across various DNA amplification methods for diagnostics [109, 112], as shown in figure 3(e). In the POC setting, we utilize collateral cleavage of CRISPR-Cas12 activity. The carefully designed crRNA binds to the target DNA, forming a ribonucleoprotein (RNP) complex with the Cas12 protein. Once the target is recognized, the activated RNP complex induces collateral cleavage of a single-stranded DNA (ssDNA) reporter molecule. This cleavage results in the emission of a fluorescence signal, which the POC device detects. This method provides a rapid, sensitive, and specific means of detection suitable for POC settings, including the potential detection of MPox.

On the other hand, Cas13 is distinguished by its unique RNA targeting mechanism, which targets ssRNA and operates with a tracrRNA and crRNA. Its high catalytic efficiency (0.96–4800 s^−1^) uses the protospacer flanking site requirement for an ‘H‘(not’G’) nucleotide adjacent to the target. Cas14, a recent addition to the CRISPR toolkit, stands out for its ability to target ssDNA without a PAM sequence [63]. So, the distinct mechanism of the Cas enzyme contributes to the broad range of applications of CRISPR, including diagnosis.

### 3.2. Cas 12-based diagnostics and its advantage

The CRISPR Cas12 system has revolutionized molecular diagnostics with its precise genome editing and innovative diagnostic capabilities [113]. Cas12 systems accurately detect specific genetic sequences, thanks to the design of programmable crRNA targeting specific genetic markers. The unique ‘collateral cleavage’ of Cas12 activity targets ss/dsDNA, pivotal in platforms like DETECTR (DNA endonuclease-targeted CRISPR trans reporter) and HOLMES (one-hour Low-cost Multipurpose highly Efficient System) for DNA-based diagnostics [17, 63, 108]. DETECTR uses Cas12a, combining DNA pre-amplification with fluorescence signaling upon target detection. HOLMES, employing PCR, has been adapted for DNA [108, 109, 114]. This interaction initiates the cleavage of ssDNA reporter (FAM-biotin or FAM-quencher) molecules that contain a quencher and fluorophore, leading to fluorescence upon successful target recognition and cleavage. The two halves of the reporter would be separated to generate detectable signals. The integration of Cas12 into POC diagnostics, exemplified by methods like NASBACC, marries isothermal amplification with CRISPR precision for rapid, specific detection [115]. CRISPR diagnostics, leveraging isothermal amplification LAMP and RPA at moderate temperatures, offer advantages in speed, specificity, and cost-effectiveness, overcoming traditional PCR method limitations of need for thermocycling [116, 117]. Cas12 minimizes false positives in isothermal amplification by requiring three specific bindings: crRNA-Cas, crRNA-target, and Cas-target via PAM, before cleavage. This technological advancement, notably the Cas12 DETECTR principle, is likely pivotal in the diagnosis of diseases like Mpox.

## 4. Cas12-based POC diagnostics for Mpox

### 4.1. Design of CRISPR-based assays specific to Mpox

The development of precise CRISPR-based assays for Mpox virus detection hinges on a series of strategic steps. It begins with the selection of a target gene, ideally located in the central region of the Mpox virus genome, to ensure the specificity of the assay. It is followed by the critical task of designing crRNA, where their binding affinity and intensity are carefully evaluated to optimize the sensitivity of the assay. The final step involves adhering to specific design guidelines for Cas12a-crRNA, a key component in enhancing the overall accuracy and reliability of the CRISPR-based diagnostic tool.

#### 4.1.1. Choose a proper gene from the center conserved region.

**Figure 4.**
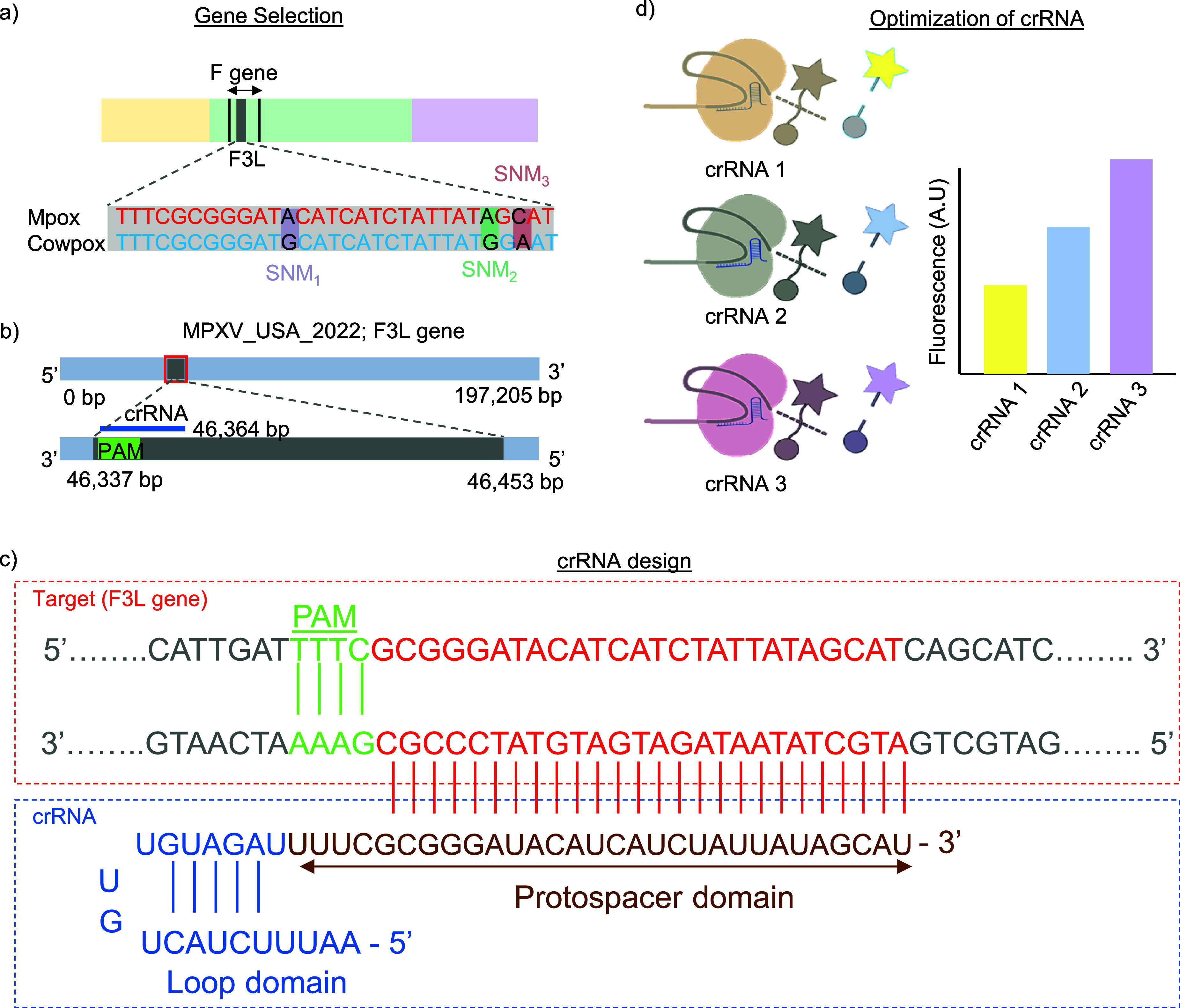
CRISPR-Cas12a Assay Design for Mpox Virus Detection. (a) Illustration of the F3L gene exclusive to the MPox virus genome, marked by a single nucleotide mismatch (SNM) distinguishing it from related orthopoxviruses like cowpox. (b) Close-up of the unique sequence of the F3L gene from the 2022 MPox strain (source Id: ON563414.3), paired with the corresponding crRNA, underscoring the protospacer adjacent motif (PAM) vital for the targeted binding of Cas12a and DNA cleavage [9, 25]. (c) Detailed structure of the crRNA, spotlighting the loop and protospacer domains that ensure high specificity of Cas12a and catalytic action. Reprinted from [120], Copyright (2021), with permission from Elsevier. (d) Visualization of signal optimization achieved through varying crRNA sequences, leading to selective cleavage of a fluorescent reporter, confirming the presence of the MPox virus.

The DETECTR strategy, leveraging the CRISPR Cas12 system, aligns well with the detection of the dsDNA-based Mpox virus. As discussed in figure 4(a), we have analyzed the complete genomic sequence of the MPox virus, focusing on the central conserved region. This region is significant due to the presence of genes encoding surface proteins, which are crucial in the transmission of genetic properties. Essential genes like DNA polymerase (E9L, G2R, E9L, F3L) are prominent in the central conserved region of the MPox virus, while D14L, J2R, and N3R are crucial in its variable region [118]. A BLAST analysis against other orthopoxviruses like cowpox, camelpox, vaccinia, and virola follows the selection of sequences from the NCBI gene bank. This analysis identified the unique single-nucleotide mismatch/mutation in the Mpox region [119], distinguishing it from orthopoxviruses like cowpox virus with A, A, and C substituting adjacent to the PAM sites. This SNP, detailed in figure 4(a), is critical in designing a crRNA that targets the MPox virus specifically. Interestingly, the trans-cleavage activity of Cas12a is notably affected by the substitution of the nucleotide A, A, and C in the seed sequence of crRNA. This particular characteristic underpins the ability of the assay to detect the MPox virus with high specificity using the Cas12a enzyme, making it an ideal target for this CRISPR-Cas12a-based cleavage assay.

#### 4.1.2. Screen and design Cas12a-crRNA and optimization.

The programmable design of crRNA is the most crucial part of the Cas12a design, which is the unique sequence for a specific target. In figures 4(b) and (c), we showed the whole designing process of crRNA from a selected target F3L gene of the MPox virus. The Cas12a crRNA consists of a single guide RNA that is 40–44 bases long, including a constant 19 nucleotide stem-loop section (loop domain) and a variable 20–24 nucleotide segment specific to the target (protospacer domain) [120]. Unique to Cas12a proteins, the loop domain plays a specific role, while the protospacer domain must be tailored to the target DNA sequence. For effective targeting, crRNAs with Cas12a locate the target DNA at sequences adjacent to the PAM, characterized by the sequence TTTV (where ‘V’ can be A, C, or G). An example of this mechanism is shown in figure 4(c), demonstrating the Cas12a/crRNA complex recognizing MPox [9]. The crRNA attaches to the DNA strand that is opposite the PAM sequence. Therefore, the protospacer domain must be precisely 20–24 bases downstream of the PAM region.

To achieve optimal target recognition in genome editing, the final step involves selecting and refining the most effective crRNA from various designs targeting a single gene. Enhanced targeting efficiency is often attained through multiple crRNAs, each designed to bind distinct sites within the gene, thereby outperforming the efficacy of a solitary crRNA. As illustrated in figure 4(d), the selection of crRNA 3, which emerged as the most effective, was based on comparative analyses of fluorescence signal intensity or gel image analysis among three distinct crRNA designs [121].

### 4.2. Sample preparation for Cas12-based detection

A critical gap in the development of effective CRISPR-based POC molecular diagnostic devices is the integration of sample preparation. From the initial collection of samples to the advanced detection of pathogens, laboratory processes in modern biological research encompass a series of intricate and crucial steps. The process begins with sample collection, which requires precision and strict protocol adherence to ensure sample integrity [122]. This stage involves collecting biological specimens, such as saliva, swabs, blood, tissue, or environmental samples, under controlled conditions to prevent contamination and degradation. Recently, the HUDSON protocol has been introduced as a method for sample preparation. It involves heating steps to deactivate nucleases and lyse viral particles in clinical samples. However, this method increases the complexity of the diagnostic process and is associated with RNA-based detection [123].

MPox samples are typically classified into two distinct collection schemes: Direct Processing and Transfer Processing. In Direct Processing, samples such as saliva, urine, semen, stool, serum, and blood are immediately prepared for centrifugation. This approach allows for the direct handling of these fluid samples without requiring intermediate steps. On the other hand, Transfer Processing is required for samples that necessitate an initial transfer to a liquid phase or additional cell lysis steps. This category includes all types of swabs and skin biopsy samples. In these cases, the samples undergo a preparatory phase to ensure they are suitable for subsequent analysis and processing.

**Figure 5.**
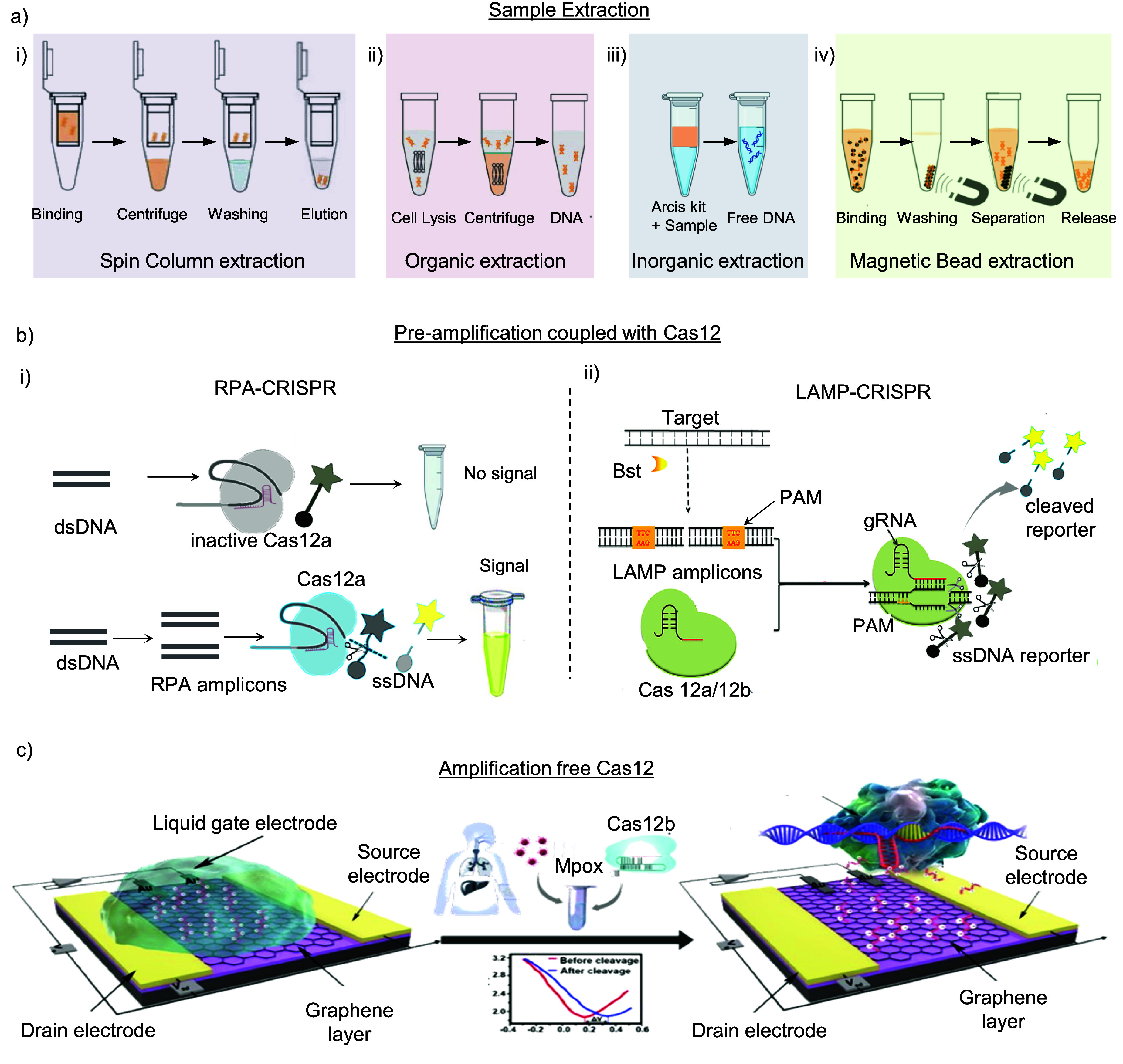
Sample extraction and pre-amplification of the sample (b) Pre-amplification assay. (i) Left section. CRISPR-Cas12a detection mechanism is featured in two scenarios: the top panel depicts the CRISPR system without RPA, where the absence of target amplification leads to no signal generation; the bottom panel illustrates the CRISPR system coupled with RPA, emphasizing the enhancement in target detection and signal amplification upon high target concentration. The accompanying inset in the bottom panel visualizes the detection process, potentially through fluorescence or alternative visual readouts. [84] John Wiley & Sons. © 2022 Wiley Periodicals LLC. (ii) Right section. The LAMP-CRISPR system, particularly Cas12a or Cas12b, is illustrated in two stages: target recognition with guide RNA (gRNA) and subsequent cleavage, followed by the collateral cleavage of a single-stranded DNA (ssDNA) reporter molecule; redrawn from the ref. Reproduced from [85]. CC BY 4.0. (c) Amplifiaction free process. Illustration of an amplification-free CRISPR-Cas12b detection system utilizing a graphene-based field-effect transistor (g-FET). The system consists of a liquid gate, source, and drain electrode, with a graphene layer forming the active sensing area. Upon introduction of the Mpox virus, the Cas12b enzyme, shown in complex with its guide RNA, targets the virus DNA. This interaction leads to cleavage of the DNA, resulting in a detectable change in the electrical signal of the FET, as depicted in the inset graph showcasing current levels before and after DNA cleavage. Reprinted from [90], with the permission of AIP Publishing.

Viral DNA separation involves four main methods: spin column isolation, organic extraction, inorganic separation, and magnetic bead purification. Spin column isolation employs membranes like glass fiber, silica derivative, or ion exchange materials for DNA trapping, with centrifugal force or vacuum for subsequent steps; it is easy to use and can be automated, though clogging from particulate matter is a potential issue [120], as shown in figure 5(a)-(i). Organic extraction mixes samples in a phenol solution, followed by centrifugation to extract DNA from the upper aqueous phase, then isolated via alcohol precipitation and rehydration [124], as depicted in figure 5(a)-(ii). While highly effective, this method is difficult to automate and requires significant manual labor. The Inorganic extraction method is both simple and swift. Arcis chemical kits have recently enabled sample extraction within 3 min from an initial volume of 30 *µ*l [16], as illustrated in figure 5(a)-(iii). In Magnetic bead purification, viral DNA binds to magnetic beads. Then, an external magnetic field is applied for stability during washing and collection. This method provides quick processing, though manual handling of particles can be labor-intensive [125, 126]. Figure 5(a)-(iv) illustrates the Magnetic bead DNA extraction process used for pre-amplification. Methods involving magnetic and spin column DNA extraction are commonly referred to as solid extraction methods. Conversely, organic and inorganic extraction methods fall under liquid DNA extraction methods [127].

The extracted material then enters the phase of molecular analysis. DETECTR strategy employing Cas12 is utilized with or without pre-amplification while maintaining specificity. Integrating the sample collection process with all subsequent steps in CRISPR detection marks a significant advancement in POC diagnosis, highlighting the evolution in current biological research methodologies.

### 4.3. Pre-amplification coupled with Cas12

Pre-amplification enhances the detection sensitivity by increasing the number of target DNA molecules before Cas12 detection, making it possible to identify Mpox even at very low initial concentrations. The process involves amplifying the specific gene of the Mpox using PCR, LAMP, and RPA, followed by the application of Cas12 for specific and sensitive detection. However, PCR techniques consume high amounts of power and energy for thermocycling [128, 129]. So, it is not recommended for POC settings for amplification. RPA and LAMP are suitable pre-amplification methods coupled with Cas12.

***RPA-CRISPR***. After extracting the viral dsDNA sample, it is amplified using RPA. This amplification makes the testing process rapid and eliminates the need for thermal cycling. After RPA, the amplicons are mixed with crRNA and Cas12a, forming a RNP complex [130]. When Cas12a encounters specific complementary DNA (cDNA), it activates and starts trans-cleavage, fragmenting ssDNAs. In the absence of the target dsDNA, like Mpox dsDNA, RNP remains inactive, leaving the ssDNA reporter intact [9]. The left panel of figure 5(b) shows the detailed mechanism. This approach offers a robust, stable room temperature and efficient method for pathogen detection.

***LAMP-CRISPR***. Similarly, the LAMP-CRISPR assay mechanism begins with extracting genomic Mpox DNA, followed by LAMP amplification, including a Cas12 PAM site for CRISPR/Cas12-based identification. In the right panel of figure 5(b), Chen *et al* Shows CRISPR/Cas12b, guided by gRNA, binds to the target sequence, activating the CRISPR/Cas12b effector for trans-cleavage activity [85].

However, LAMP-CRISPR is less suited for POC applications because it needs two separate temperature steps for LAMP and CRISPR reactions. In contrast, RPA-CRISPR operates at a uniform temperature, making it more energy-efficient. This has led to the predominant use of the RPA amplification technique in CRISPR-based pre-amplification assays. Significantly, pre-amplification adds sensitivity to the assay while incorporating CRISPR enhances its specificity.

### 4.4. Amplification free detection

Amplification-free CRISPR detection is necessary for rapid, onsite diagnostic tests without complex laboratory infrastructure. Most CRISPR-based diagnostics employing Cas enzymes without prior target amplification typically report a LOD in the picomolar range [115]. It enables the direct identification of nucleic acids from samples. Amplification-free techniques can involve utilizing graphene field-effect transistor (g-FET) biosensors [90], optimizing crRNAs and signal reporters for the CRISPR/Cas system [20], using digital droplet-based detection [131], employing signal transducers like fluorometric and SERS-based sensors [92, 93], and incorporating cascade signal amplification methods [132].

The principle of the g-FET biosensor, when combined with the Cas12b system, is to create a platform for rapid, amplification-free detection of the mpox virus. It is achieved by immobilizing ssDNA reporters on a graphene surface within the gFET. When Mpox DNA is introduced, it interacts with the Cas12b–sgRNA complex, activating the nuclease activity of Cas12b, which cleaves the ssDNA reporters. This cleavage shifts the electrical current and voltage of the g-FET, resulting in a detectable sensor signal output, illustrated in figure 5(c). This CRISPR-gFET system is sensitive, rapid, and does not require prior amplification of the DNA, making it a promising tool for POC diagnostics. The collateral cleavage of Cas13 on a graphene surface enables SARS-CoV-2 detection with 1 aM sensitivity in just 30 min [90, 133]. However, FET biosensors for POC diagnostics face limitations in robust bio-element immobilization, consistent mass production for reliability, and high selectivity to prevent false positives [134].

Linking Cas effectors with signal amplification (cascade signal amplification), like the Csm6 enzyme, improves the LOD in amplification-free assays. A notable example is coupling LbuCas13a with the Csm6 variant from Thermus thermophilus, which boosted the sensitivity for detecting SARS-CoV-2 RNA beyond what Cas13 alone could achieve. However, the sensitivity of diagnostic methods using the Cas effector and Csm6 cascade is limited to around 500 fM to 1 nM RNA, translating to roughly 10^5^–10^9^ copies *µ*l^−1^. This level of sensitivity is achieved without incorporating RT-LAMP to amplify the target sequence [135].

Digital droplet CRISPR is a method that segments reactions into micro-units, enhancing the sensitivity and specificity of biomedical analysis. The technology includes submerging water in oil and passive fluid distribution by microchannel geometry. It requires small sample volumes and offers rapid reaction speeds. Digital droplet is integrated with Cas12 and microchamber array technologies for amplification-free, ultra-sensitive biosensing [131]. The system can detect targets in minutes and is more sensitive than other amplification-free platforms. Politza *et al* reported that amplification-free digital CRISPR attains a LOD of 1.6–2.4 aM [63]. Additionally, the CRISPR-Chip offers a swift method for detecting genomic DNA without pre-amplification. However, pre-amplification can enhance its sensitivity, though this comes with higher resource usage and added complexity [136]. However, the sensitivity of such systems may vary depending on droplet size and the optimization of CRISPR components like crRNAs.

The principle of Optical Sensing based on Surface Plasmon Effects, particularly in the context of SERS strategies coupled with Cas12a, involves leveraging the enhanced electric field on metal surfaces. When external electromagnetic radiation interacts with free electrons in nanostructures, immobilized molecule probes on plasmonic nanostructures generate intense Raman signals. This ultrasensitive detection capability is attributed to surface plasmon effects. SERS-integrated CRISPR/Cas biosensors utilize these intense Raman signals, altered by the fragmentation of bound single-strand probes upon Cas12a-mediated trans-cleavage, for nucleic acid biosensing. This method has achieved remarkable detection levels, down to fM concentrations, in minimal time [132]. Enhancements using nanomaterials can improve detection to aM levels.

Electrochemiluminescence sensors integrated with Cas12a systems attach electrochemical tags like methylene blue to electrode surfaces through nucleic acid strands. When CRISPR/Cas complexes, activated by the target nucleic acid, cleave these strands, the distance between the tags and the electrode surface changes. This change alters the electrochemical signal by reducing the current peak or varying the electron transfer rate. This signal alteration is used to detect nucleic acids with high sensitivity, sometimes enhanced by additional components like nanoparticles or specific aptamers.

While these advanced diagnostic techniques have not yet been applied to Mpox virus detection, they have been successfully used to identify other targets. These include African Swine Fever Virus, Epstein–Barr virus, Hepatitis B Virus, various strains of Human papillomavirus (HPV, HPV-16, HPV-18), SARS-CoV-2, Human Immunodeficiency Virus (HIV-1), and Influenza viruses (PB-19) dsDNA. These diverse applications showcase their potential to detect various pathogens [132].

### 4.5. Detector and overall testing process for Cas 12 assay

In the realm of CRISPR-Cas12-based assays, a suite of transduction methods offers advantages and potential drawbacks. Fluorescence detection stands out for its sensitivity and is adept at real-time monitoring. The method uses fluorescence changes, like Förster resonance energy transfer (FRET) [22], to signal biorecognition events. While this amplification enhances specificity, it may not meet clinical-level, amplification-free detection requirements and can suffer from background noise and photobleaching [137]. Colorimetric methods for Cas12 detection, such as LFA and plasmonic nanoparticle-based assays, offer simple, rapid, and cost-effective diagnostics [138]. LFAs use gold nanoparticle-conjugated antibodies to visually indicate the presence of a target via color change [139]. Plasmonic nanoparticles, on the other hand, utilize the localized surface plasmon resonance phenomenon, where CRISPR-mediated target recognition alters interparticle distances, causing a detectable color shift [140]. For example, a paper-based sensor can differentiate Zika from Dengue via a color shift from yellow to purple, indicating the presence of target RNA [141]. This method, paired with amplification techniques like NASBA, achieves femtomolar sensitivity [115]. Colorimetry is quick and user-friendly, offering direct visual readout, but it falls short in sensitivity and can have a higher error rate. Bioluminescence or Chemiluminescence is noted for its high signal-to-noise ratio, yet additional reagents can complicate the assay. Gel electrophoresis provides direct visual results at a lower cost. Still, it is generally restricted to laboratory environments due to its operational requirements, time-consuming, non-quantative to detect targets, and limited resolving power for small DNA fragments [132].

**Figure 6.**
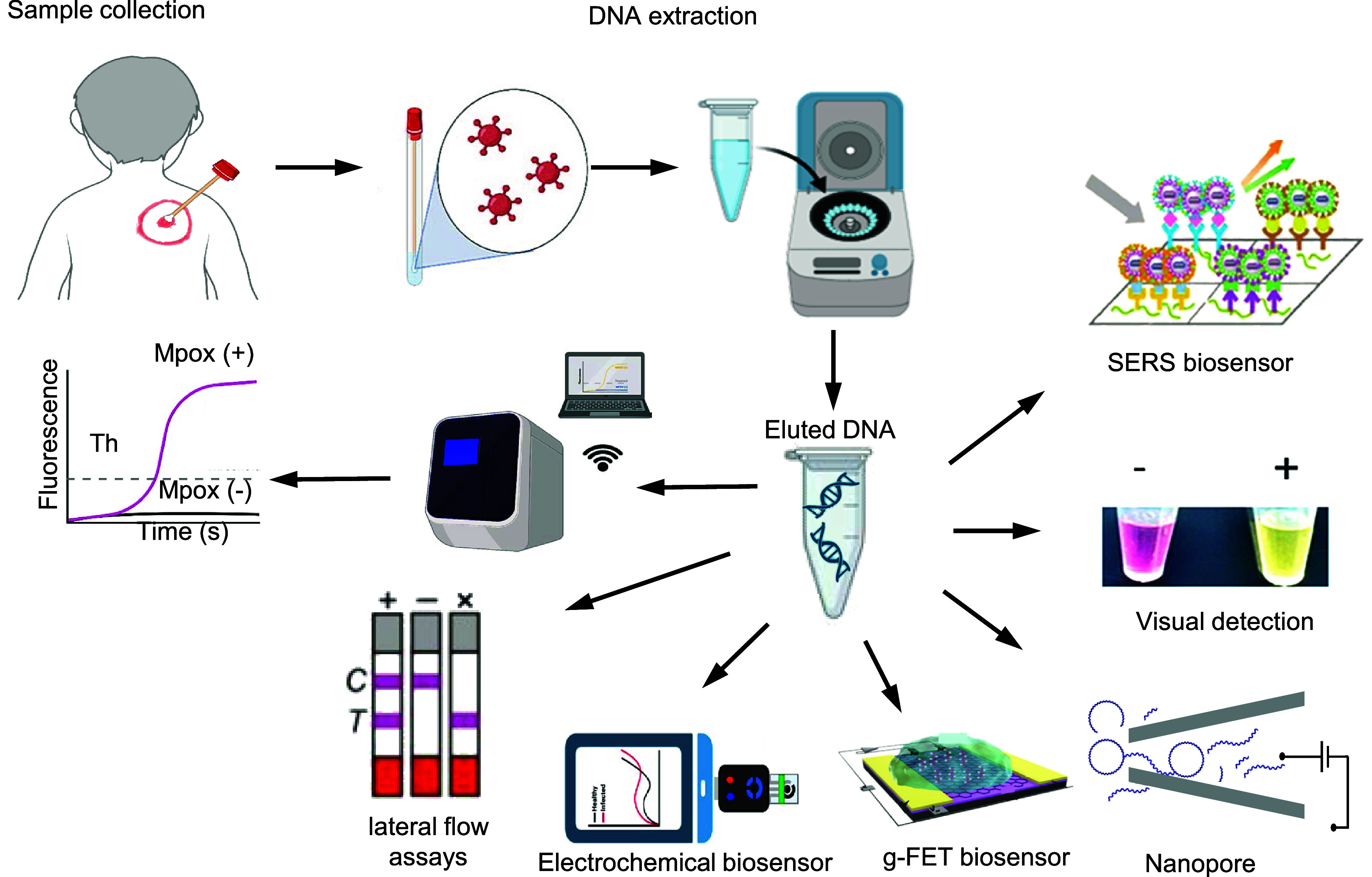
Overall testing process for Mpox detection using cas12, starting from sample collection from a patient showing targeted symptoms. The process includes sample preparation, isolation of viral dsDNA, and application of various diagnostic techniques, such as colorimetric analysis and lateral flow assays (LFA). Advanced methods like nanopore sensors, electrochemical sensors, and SERS biodevices are also depicted. The figure illustrates the transition from clinical sampling to laboratory analysis, such as PCR amplification, concluding with the readout of results, which may involve visual or electronic data representation. Figures are drawn using Biorender and reference used in the text.

SERS and refractive index methods promise ultra-high sensitivity and label-free detection, respectively, but demand high-end instrumentation [142]. Electrochemical sensors are well-established for their sensitivity, but background noise can be an issue [143]. The g-FET impresses with ultra-high sensitivity and real-time data acquisition yet is complex in fabrication and sensitive to background interference [144]. The details of the electrochemical sensor, SERS, and g-FET are discussed in section 4.4. Ahamed *et al* recently employed a nanopore sensor integrated with RPA-CRISPR technology, successfully detecting the Mpox virus with a Lod 16 copies *µ*l^−1^ [9]. Nanopore sensors are notable for their high sensitivity and monitoring capabilities but have lower throughput and can suffer from background noise [145]. Figure 6 summarizes the overall detection process and most established detection methods. Conductivity methods offer the convenience of low instrument requirements; however, their lower sensitivity and changes in permselectivity behavior can restrict their effectiveness in detecting targets with low abundance [146]. While electronic, fluorescence, and g-FET methods have been explored for Mpox detection, many other techniques still offer significant research opportunities. These unexplored methods could expand the diagnostic toolkit for Mpox, building on their success in detecting other contagious pathogens like SARS-CoV-2, HIV, and Dengue fever.

### 4.6. Current Cas12-enhanced portable device for Mpox detection

**Figure 7.**
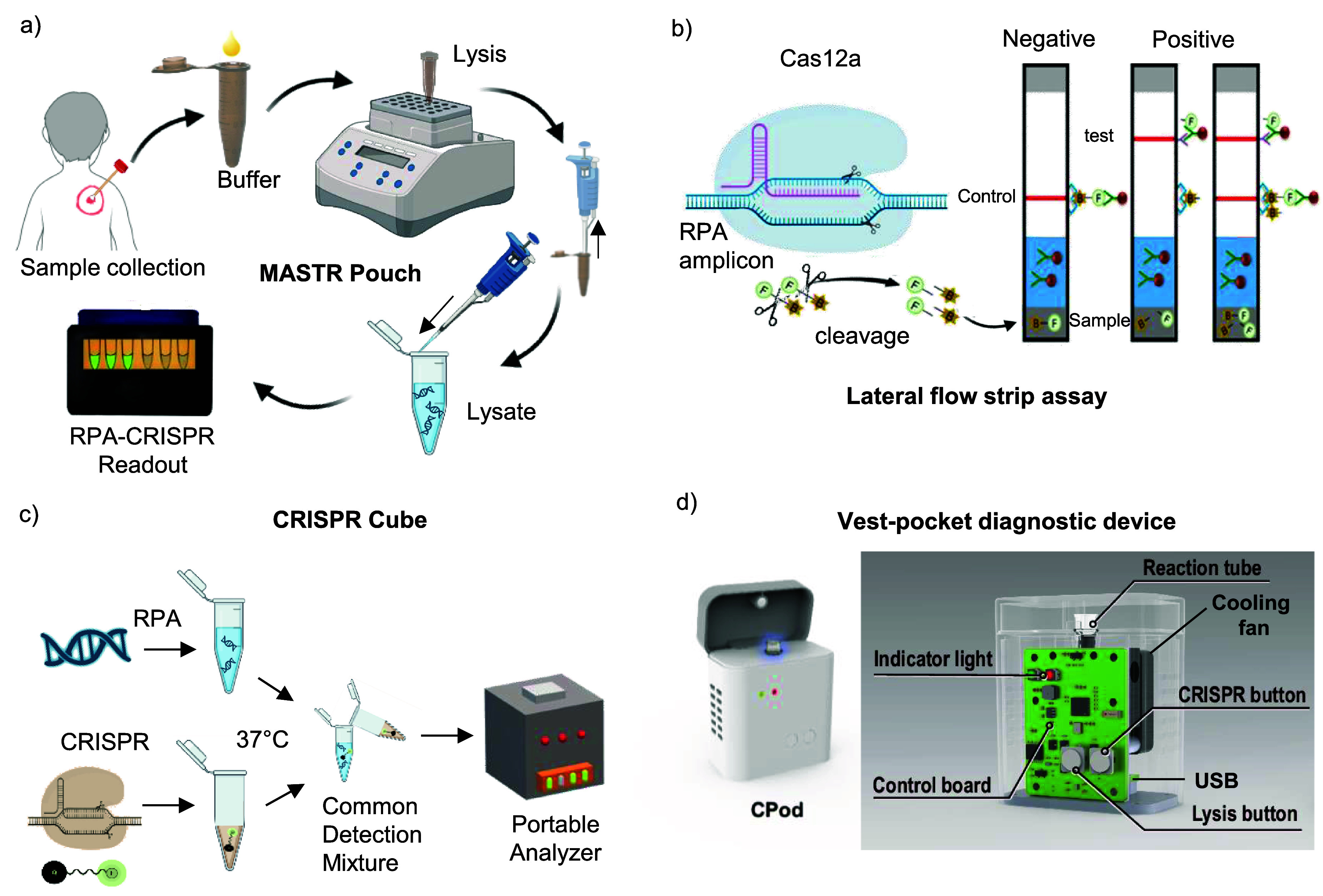
Overview of the current diagnostic workflow utilizing CRISPR technology for MPox detection. (a) MASTR Pouch (Mpox At-home Self-Test and point-of-caRe Pouch): The detection of the Mpox virus is facilitated by a straightforward process using the compact, palm-sized MASTR Pouch device; redrawn from ref. Reprinted from [87], Copyright (2023), with permission from Elsevier. (b) Lateral flow strip (LFS) assay: The RAA method is utilized for amplifying the DNA template, the CRISPR/Cas12a system for cleaving the reporter, and both fluorescence and lateral flow strip assays serve as the means for detection signal output; redrawn from ref. Reproduced from [147] with permission from the Royal Society of Chemistry. (c) CRISPR Cube: Amplification of target DNA, using precise temperature control to increase the quantity of the target DNA and interpret the signal; redrawn from ref. [82] John Wiley & Sons. © 2023 Wiley Periodicals LLC. (d) Portable CRISPR-SPR-FT biosensing platform: The Mpox detection platform uses a design where AuNPs are bound to ssDNA reporters on a biosensor; these reporters are cleaved by Cas12a–crRNA upon encountering the target DNA. The cleavage results in a change in the surface plasmon resonance (SPR) signal due to the detachment of AuNPs, with this change being captured and recorded in real-time by a fiber-optic system, ensuring target specificity by differentiating among various dsDNA concentrations, redrawn from ref. Reproduced from [89], with permission from Springer Nature.

In recent years, the application of CRISPR technology in infectious disease diagnostics has seen significant advancements, particularly in the detection of Mpox. The comprehensive analysis of recent studies in POC that have employed CRISPR-based methods is discussed here. Recently, the MASTR Pouch (Mpox at-home self-test and POC Pouch) device was developed to detect the Mpox virus. In figure 7(a), this step in the diagnostic sequence initiates the collection of a mock pseudo sample, likely to contain viral particles if the subject is infected. Following collection, the sample is introduced to a buffer solution within the MASTR Pouch, which facilitates the lysis or breakdown of viral particles, releasing their DNA. The liberated viral DNA lysate is then processed for amplification and detection. The final stage of this diagnostic flow involves the RPA-CRISPR readout, where a fluorescence signal indicates the presence of the virus, concluding the process from sample collection to obtaining a visible result [87]. In figure 7(b), the LFS assay depicted operates based on the RAA-Cas12a MPox detection method. This process involves using a reporter molecule (FB reporter) modified with 6-FAM at the 5‘end and biotin at the 3’ end. The assay begins with incubation at 37 °C for 40 min [147]. Upon completion, sterile deionized water is added to the reaction to prepare it for application to the LFS. After a further 5 min of incubation at room temperature, the appearance of a color band on the strip indicates a positive result for the presence of the Mpox virus, while its absence, leaving only the control band colored, indicates a negative result [82].

The CRISPR Cube device utilizes the distinct DNA-cleavage properties of Cas12a to detect specific genes, such as the F3L gene of the MPox. This platform, mentioned in the study by Singh *et al* employs a diagnostic kit that leverages orthogonal CRISPR-Cas12a trans-cleavage activity for the simultaneous detection of multiple viral genes, overcoming the challenge often faced in multi-gene detection with CRISPR technology, as illustrated in figure 7(c) [82]. Chen *et al* developed an ultra-sensitive CRISPR-surface plasmon resonance based Fiber tip biosensing platform for detecting Mpox DNA. This compact, SPR-based fiber tip biosensor can sense changes in the surface load with extreme sensitivity, employing the high specificity of the CRISPR/Cas12a system to target DNA. Chen *et al* designed the biosensor to be decorated with gold nanoparticles (AuNPs) linked through partially cDNAs that include an ssDNA reporter, thereby enabling the precise detection of target DNA. The innovative combination of the specificity of CRISPR/Cas12a with the ultrasensitive detection ability of the SPR forms the basis of this portable biosensing platform [81]. Wang *et al* invented a vest pocket so-called Streamlined CRISPR On Pod Evaluation platform device that can use for field deployability and detection of 2.5 copies/reaction Mpox virus in 15 min from sample to answer, as shown in figure 7(d) [89].

Recent studies have demonstrated the potential of CRISPR technology as a powerful tool for the rapid and specific diagnosis of Mpox. It utilizes its programmable crRNA to identify viral DNA with high accuracy. The integration of CRISPR with advanced diagnostic platforms, such as LFAs and SPR-based fiber tip biosensors, has enhanced the sensitivity and speed of Mpox detection, facilitating POC testing. These advancements underscore the transformative impact of CRISPR in infectious disease diagnostics, paving the way for more effective surveillance and management of Mpox outbreaks.

### 4.7. Comparative effectiveness with conventional diagnostic approaches

The development of CRISPR-based POC diagnostics for Mpox represents a significant leap forward in infectious disease management, particularly compared to conventional diagnostic methods. Unlike traditional approaches such as PCR and virus isolation, which require sophisticated laboratory equipment and extended processing times, CRISPR-based POC diagnostics offer rapid, accurate, and on-site testing capabilities [89]. This innovative method harnesses the precision of CRISPR technology to detect specific genetic sequences of the Mpox virus, enabling quicker diagnosis than PCR, which is time-intensive and less feasible in resource-limited settings. Furthermore, CRISPR diagnostics overcomes the limitations of culture-based methods, which are slow and require high biosafety levels [148]. By providing results in real-time, CRISPR-based POC diagnostics not only facilitate immediate clinical decisions and public health interventions but also reduce the risk of false negatives inherent in less sensitive conventional methods [149]. The comparative effectiveness of CRISPR-based diagnostics lies in their potential to transform Mpox surveillance and response strategies, especially in outbreak scenarios and regions lacking advanced laboratory infrastructure, thereby playing a crucial role in global health security. Table 2 provides a comprehensive summary of CRISPR-based POC methods for detecting Mpox, detailing their LOD across various amplification techniques and detection methods.

## 5. Challenges, opportunities, and outlook for CRISPR-based Mpox detection

This review focuses on integrating CRISPR-Cas12 into POC devices for Mpox virus detection, facilitating a seamless ‘sample-to-answer’ process. However, the development and implementation of CRIPSR-based diagnostics face several technical and practical challenges, particularly in streamlining processes for POC applications. These challenges include integrating complex laboratory processes into user-friendly formats, ensuring the stability and effectiveness of CRISPR components outside controlled environments, and adapting the technology for diverse and often resource-limited settings [150]. Additionally, the need for multiplexing capabilities and efficient field-deployable systems underscores the demand for advanced yet accessible diagnostic solutions. Additionally, the need for multiplexing capabilities and efficient field-deployable systems underscores the demand for advanced yet accessible diagnostic solutions [151]. Addressing these issues is crucial for leveraging the full potential of CRISPR-based diagnostics in various clinical and field settings. The ongoing research and innovation in this field aim to overcome these hurdles, paving the way for broader adoption and impact of CRISPR technology in healthcare.

**Figure 8.**
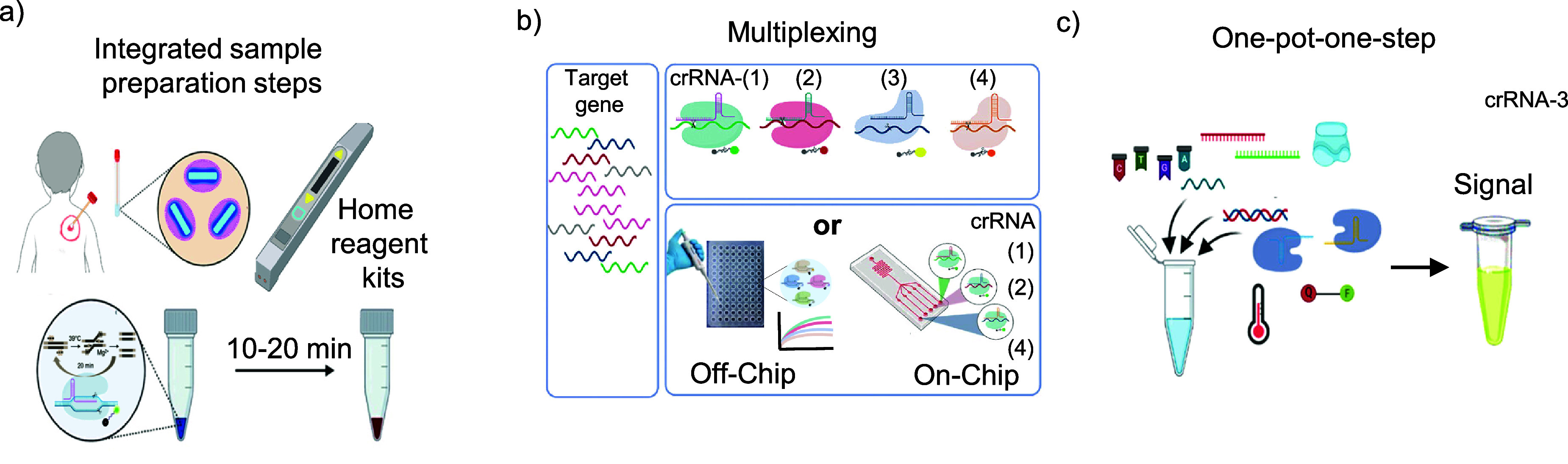
Future Challenges and Opportunities. (a) Integrated sample preparation steps inside the device. (b) Multiplexing of MPox for various variants. [150] John Wiley & Sons. © 2023 The Authors. Advanced Science published by Wiley-VCH GmbH (c) Developing one-pot-one-step assays for POC settings; redrawn from ref. Reprinted with permission from [155]. Copyright (2022) American Chemical Society.

***Integrated sample preparation steps (sample to answer)***: Integrated sample preparation in portable devices is vital for POC testing, allowing for rapid, on-site diagnostics, especially in low-resource settings [152]. These devices streamline the complex sample preparation process traditionally required for biochemical and molecular assays like ELISA and PCR [153]. Current technologies, like chip or cartridge systems and paper-based microfluidics, enable this integration. Figure 8(a) illustrates the possible combination of integrated sample preparation steps. However, challenges include simplification to match lab-quality processing, ensuring device adaptability to various sample types, and maintaining cost-effectiveness. Liu *et al* *developed* a semi-automated POC device for HIV viral load testing using whole blood samples [154]. However, this device does not incorporate integrated sample preparation steps. Addressing these challenges involves leveraging advances in nanomaterials, microfluidics, and portable power sources, which can lead to innovations like photonic lysis and ultrafast amplification. The goal is to produce robust, sensitive, and specific POC devices that are easy to use and manufacture, even for complex clinical samples.

***Multiplexing in diagnostics***: Multiplexing is a crucial aspect of CRISPR-based diagnostics, allowing for the simultaneous detection of multiple disease targets and the differentiation of various pathogenic strains. This capability is vital for a comprehensive syndromic approach to molecular diagnostics, as it provides more extensive data for guiding treatment. Figure 8(b) shows the multiplexing and signal detection concept. Although platforms like BioFire effectively detect a range of respiratory pathogens in one sample, limitations persist in concurrently identifying all variants [150]. CRISPR diagnostics using Cas13 effectors are promising for POC multiplexing, leveraging the specificity of enzymes for specific dibase sequences. Innovations such as SHERLOCK.V2 and OPTIMA-Dx have made strides in this area, employing various Cas effectors and thermostable enzymes for effective multiplexing in a single reaction. However, challenges in extending this multiplexing capacity beyond four targets, particularly in systems like SHERLOCK.v2, are evident due to pre-amplification constraints and the complex primer requirements in LAMP pre-amplification [156]. Tombuloglu *et al* developed a multiplex RT-PCR method for early and efficient detection. The designed assay simultaneously detects two viral genes, N and RdRP, and a human gene, RP, ensuring fast, reliable, and high-throughput testing, critical for controlling the disease spread [157]. Future advancements, including amplification-free modalities and high-throughput testing like the Combinatorial Arrayed Reactions for Multiplexed Evaluation of Nucleic acids approach, hold the potential to expand significantly the multiplexing capabilities of CRISPR-based diagnostics, which is particularly valuable during pandemics [158]. However, in the case of Mpox, there has been limited exploration of multiplexing for differentiating between various variants.

***One-Pot-One-Step approach***. A significant advancement in CRISPR-based diagnostics is the development of ‘one-pot-one-step’ assays, where sample processing, detection, and readout are combined into a single tube. This approach simplifies the workflow and reduces the time from sample to result. Innovations like the colorimetric RT-LAMP assay for SARS-CoV-2 detection exemplify this, combining the lysis and detection steps in a single reaction. Challenges persist in the ‘one-pot-one-step’ CRISPR assays, particularly in maintaining high sensitivity and specificity while integrating the diagnostic process. Overcoming issues with compatibility and cross-reactivity remains a crucial challenge to ensuring reliable assays [150]. Figure 8(c) shows the concept of a one-pot-one-step reaction for Mpox detection. Recently, most of the research on RPA-CRISPR has been carried out by putting one of the reagents on the lid and another on the bottom [155, 159]. They require extra centrifugation force to combine and start the cleavage activity of Cas12. Uno *et al* developed a CRISPR gel biosensing platform for rapid HIV RNA detection, integrating CRISPR-Cas12a within an agarose gel. This gel acts as an interface for the RT-RPA reaction, allowing initial amplification followed by CRISPR-mediated detection [159]. Xun *et al* designed the SPOT assay, a rapid testing method utilizing a wax barrier to separate CRISPR and LAMP reagents. The wax melts by heating to over 70 °C, allowing the components to mix, thus facilitating the reaction in a single container, though not in a single uninterrupted step [160]. Yan *et al* achieved the detection of miRNAs, specifically miR-21, miR-196a, miR-451a, and miR-1246, in extracellular vesicles with impressive sensitivity, identifying concentrations in the single-digit femtomolar range and demonstrating single-nucleotide specificity [161]. Despite this breakthrough, the one-pot-one-step diagnostic approach remains under active investigation.

***Field-Deployable Lyophilization***: Lyophilization, or freeze-drying, is critical in addressing the long-term storage and transport challenges of CRISPR reagents. One of the primary concerns is the dependency on a cold chain for storing and transporting CRISPR components like guide RNAs and Cas enzymes, which require ultralow temperatures to remain stable. This requirement significantly increases logistical and storage costs and poses a significant limitation in areas with limited resources. Moreover, both proteins and reagents in solution are prone to degradation—proteins can suffer from physical and chemical degradation, leading to a reduced efficacy of the reagents over time. The complexity in sample preparation also increases, as the liquid state of reagents necessitates precise measurement and mixing at the point of use, requiring skilled personnel and raising the likelihood of errors. The handling and transportation of liquid CRISPR reagents are fraught with risks of spillage and contamination, demanding stringent handling precautions and limiting their accessibility, especially in remote or resource-poor settings. Lastly, the preparation of assays from liquid reagents is time-consuming, adding to the overall time required to set up and run the diagnostic tests. These challenges collectively hinder the widespread adoption and effectiveness of CRISPR-based diagnostic tools in various settings. For LAMP coupled with CRISPR, Lyo-ready reagents, optimized for efficient lyophilization, are being developed to make CRISPR-based diagnostics more robust and field-deployable. RPA reagents are readily available in a lyophilized format, simplifying the process of coupling them with CRISPR to create robust, lyophilized RPA-CRISPR assays. This development is part of ongoing research focused on enhancing lyophilization techniques. The reagents are specifically designed with minimal antifreeze agents and pre-optimized for lyophilization. This advancement significantly reduces the reliance on cold-chain transport and extends the shelf life of these diagnostics, making them more viable for use in low-resource and remote settings.

Looking ahead, CRISPR-based detection systems like CRISPR-Cas12 hold immense potential for revolutionizing Mpox diagnostics. These systems promise POC testing, integration with smartphone technology for rapid data analysis, and multiplexing capabilities to detect various pathogens simultaneously. The evolution towards more automated and AI-integrated systems could further improve diagnostic accuracy and efficiency. The diagnostic strategy for Mpox, utilizing a combination of RPA and CRISPR-Cas12, provides improved accessibility, accuracy, and comprehensive disease monitoring, a crucial advancement for areas with limited resources [162]. However, future breakthroughs in CRISPR diagnostics are expected to enhance sensitivity and specificity, enable multiplexing of viral mutations, and integrate one-pot assays with amplification for practical POC applications.

## Data Availability

No new data were created or analysed in this study.
